# Supplementary material for: Land use and pollinator dependency drives global patterns of pollen limitation in the Anthropocene
Source: Nat Commun. 2020 Aug 10;11:3999. doi: 10.1038/s41467-020-17751-y (PMC7417528; doi:10.1038/s41467-020-17751-y)
Supplement: Supplementary file 1 — Supplementary Information [file 41467_2020_17751_MOESM1_ESM.pdf]

Land use and pollinator dependency drives global patterns of pollen limitation in the Anthropocene

Bennett et al

Supplementary Table 1. Sample size (K), bootstrapped model estimates of pollen limitation and upper and lower confidence intervals from 500 phylogenetic meta-analyses with the interaction between major land use type (classified as either natural, managed or urban) and pollinator dependence (classified as either autofertile or pollinator dependent) as predictors and pollen limitation as the response variable. Random effects include phylogeny and experimental design. Source code and data are archived on github see data and code availability statements.

Land use x pollinator dependence	K	Estimate	Lower CI	Upper CI
Natural: Pollinator dependent	1097	1.01	0.567	1.486
Managed: Pollinator dependent	584	0.910	0.403	1.39
Urban: Pollinator dependent	70	1.43	0.344	2.65
Natural: Autofertile	251	-0.213	-1.17	0.936
Managed: Autofertile	183	-0.190	-1.08	0.953
Urban: Autofertile	13	-0.686	-4.22	0.814

Supplementary Table 2. Results from a phylogenetic meta-analysis with the interaction between major land use type (classified as either natural, managed or urban) and pollinator dependence (classified as either autofertile or pollinator dependent) as predictors and pollen limitation as the response variable, with *a priori* contrasts conducted between pollinator dependence and land use using the Holm adjustment for multiple comparisons. All tests were two-sided. Random effects include phylogeny and experimental design. Source code and data are archived on github see data and code availability statements.

Land use x pollinator dependence		df	QM	P-value
		6	13294	<0.001
Contrasts	Estimate	SE	t-value	P-value
Natural: Pollinator dependent - Managed: Pollinator dependent	0.134	0.008	17.8	<0.001
Managed: Pollinator dependent - Urban: Pollinator dependent	-0.723	0.015	-48.7	<0.001
Natural: Pollinator dependent - Urban: Pollinator dependent	-0.589	0.014	-41.6	<0.001

Supplementary Table 3. Bootstrapped model estimates and upper and lower confidence intervals from 500 phylogenetic meta-analyses with the interaction between major land use type and ecological specialisation (classified as pollinated by either one, few or many pollinators) as predictors and pollen limitation as the response variable. Random effects include phylogeny and experimental design. Source code and data are archived on github see data and code availability statements.

Land use x ecological specialisation	K	Estimate	Lower CI	Upper CI
Natural: One	107	1.51	0.465	3.08
Managed: One	41	1.47	0.670	2.46
Natural: Few	359	0.992	0.304	1.78
Managed: Few	177	0.727	0.061	1.44
Natural: Many	363	0.661	0.137	1.14
Managed: Many	174	0.669	0.189	1.11

Supplementary Table 4. Bootstrapped model estimates of pollen limitation and upper and lower confidence intervals from 500 phylogenetic meta-analyses with the interaction between major land use type and functional specialisation (classified as plants pollinated exclusively by bees, exclusively by any other functional group or pollinated by multiple functional groups) as predictors and pollen limitation as the response variable. Random effects include phylogeny and experimental design. Source code and data are archived on github see data and code availability statements.

Land use x functional specialisation	K	Estimate	Lower CI	Upper CI
Natural: exclusively bee	403	1.06	0.147	1.97
Managed: exclusively bee	183	0.663	-0.050	1.429
Natural: exclusively other	139	0.739	0.073	1.52
Managed: exclusively other	106	0.997	0.232	1.61
Natural: multiple functional groups	442	0.347	-0.241	0.882
Managed: multiple functional groups	211	0.437	-0.064	0.903

Supplementary Table 5. Results from a phylogenetic meta-analysis with the interaction between major land use type and functional specialization as predictors and pollen limitation as the response variable, with *a priori* contrasts conducted between functional specialization and land use using the Holm adjustment for multiple comparisons. All tests were two-sided. Random effects include phylogeny and experimental design. Source code and data are archived on github see data and code availability statements.

Land use × functional specialization		df	QM	P-value
		6	4518	<0.001
Contrasts	Estimate	SE	t-value	P-value
Natural: Exclusively bee pollinated - Managed: Exclusively bee pollinated	0.591	0.020	29.4	<0.001
Natural: Exclusively pollinated other - Managed: Exclusively pollinated other	-0.156	0.061	-2.58	0.010
Natural: Multiple functional groups - Managed: Multiple functional groups	-0.083	0.011	-7.34	<0.001

Supplementary Table 6. Results from a phylogenetic meta-analysis with the major land use type, pollinator dependence and their interaction as predictors and pollen limitation as the response variable. Estimates of the variance components of the random effects included in the model phylogeny and experimental design are also shown. Source code and data are archived on github see data and code availability statements. All tests were two-sided.

Land use x pollinator dependence		df	QM	P-value
		5	13294	<0.001
	Estimate	Lower CI	Upper CI	P-value
intercept	-0.531	-8.08	7.02	0.890
Pollinator dependent	1.56	1.53	1.59	<0.001
Natural	-0.050	-0.067	-0.034	<0.001
Urban	-0.104	-0.194	-0.015	0.023
Natural: Pollinator dependent	0.185	0.163	0.206	<0.001
Urban: Pollinator dependent	0.827	0.734	0.921	<0.001
Random effects	Estimate			
Design	0.475			
Phylogeny	46.7			

Supplementary Table 7. Results from a phylogenetic meta-analysis with the major land use type, pollinator dependence and their interaction as predictors and pollen limitation as the response variable. Estimates of the variance components of the random effects included in the model phylogeny, Tau^2 and experimental design are also shown. Source code and data are archived on github see data and code availability statements. All tests were two-sided.

Land use x pollinator dependence		df	QM	P-value
		5	88.0	<0.001
	Estimate	Lower CI	Upper CI	P-value
intercept	0.289	-0.837	1.42	0.615
Pollinator dependent	0.393	0.266	0.520	<0.001
Natural	0.091	-0.053	0.235	0.214
Urban	-0.408	-0.801	-0.015	0.042
Natural: Pollinator dependent	-0.082	-0.244	0.079	0.318
Urban: Pollinator dependent	0.678	0.243	1.11	0.002
Random effects	Estimate			
Design	0.023			
Phylogeny	1.016			
Tau^2	0.325			

Supplementary Table 8. Results from a phylogenetic meta-analysis with the major land use type, ecological specialisation and their interaction as predictors and pollen limitation as the response variable. Estimates of the variance components of the random effects included in the model phylogeny and experimental design and without Tau2 are also shown. Source code and data are archived on github see data and code availability statements. All tests were two-sided.

Land use x ecological specialisation		df	QM	P-value
		5	6134	<0.001
	Estimate	Lower CI	Upper CI	P-value
intercept	0.563	-6.44	7.57	0.875
One	1.21	1.12	1.31	<0.001
Many	0.039	-0.018	0.096	0.176
Natural	0.383	0.334	0.433	<0.001
One: Natural	0.121	0.022	0.219	0.016
Many: Natural	-0.397	-0.451	-0.344	<0.001
Random effects	Estimate			
Design	0.453			
Phylogeny	39.2			

Supplementary Table 9. Results from a phylogenetic meta-analysis with the major land use type, ecological specialisation and their interaction as predictors and pollen limitation as the response variable. Estimates of the variance components of the random effects included in the model phylogeny, Tau^2 and experimental design are also shown. Source code and data are archived on github see data and code availability statements. All tests were two-sided.

Land use x ecological specialisation		df	QM	P-value
		5	5.26	0.385
	Estimate	Lower CI	Upper CI	P-value
intercept	0.653	-0.568	1.875	0.295
One	0.209	-0.041	0.458	0.102
Many	0.030	-0.143	0.204	0.733
Natural	0.083	-0.070	0.236	0.288
One: Natural	-0.311	-0.613	-0.010	0.043
Many: Natural	-0.121	-0.328	0.086	0.253
Random effects	Estimate			
Design	0.038			
Phylogeny	1.16			
Tau^2	0.325			

Supplementary Table 10. Results from a phylogenetic meta-analysis with the major land use type, functional specialisation and their interaction as predictors and pollen limitation as the response variable. Estimates of the variance components of the random effects included in the model phylogeny and experimental design and without Tau2 are also shown. Source code and data are archived on github see data and code availability statements. All tests were two-sided.

Land use x functional specialisation		df	QM	P-value
		5	4518	<0.001
	Estimate	Lower CI	Upper CI	P-value
Intercept	0.867	-6.09	7.82	0.807
Bee specialized	0.406	0.312	0.500	<0.001
Generalist	-0.618	-0.711	-0.525	<0.001
Managed	0.156	0.037	0.275	0.010
Managed: Bee specialized	-0.747	-0.870	-0.624	<0.001
Managed: Generalist	-0.073	-0.193	0.047	0.233
Random effects	Estimate			
Design	0.696			
Phylogeny	39.2			

Supplementary Table 11. Results from a phylogenetic meta-analysis with the major land use type, functional specialization and their interaction as predictors and pollen limitation as the response variable. Estimates of the variance components of the random effects included in the model phylogeny, Tau^2 and experimental design are also shown. Source code and data are archived on github see data and code availability statements. All tests were two-sided.

Land use x functional specialisation		df	QM	P-value
		5	4.57	0.471
	Estimate	Lower CI	Upper CI	P-value
Intercept	0.663	-0.520	1.85	0.272
Bee specialized	0.008	-0.167	0.183	0.927
Generalist	-0.070	-0.233	0.093	0.401
Managed	0.079	-0.130	0.288	0.460
Managed: Bee specialized	-0.175	-0.429	0.078	0.175
Managed: Generalist	-0.027	-0.267	0.214	0.827
Random effects	Estimate			
Design	0.034			
Phylogeny	1.10			
Tau^2	0.320			

PRISMA 2009 Flow Diagram

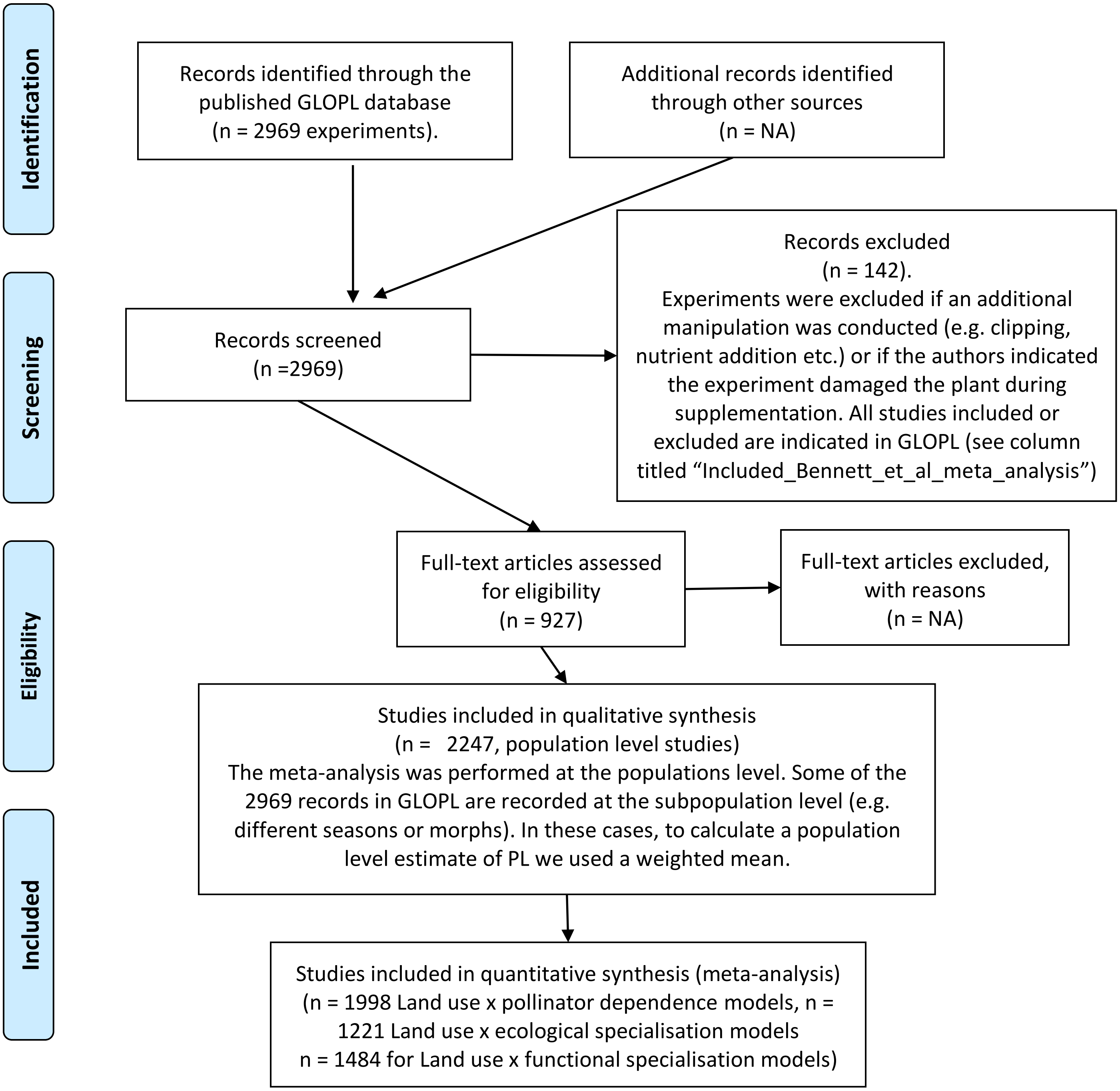

From: Moher D, Liberati A, Tetzlaff J, Altman DG, The PRISMA Group (2009). *P*referred *R*eporting *I*tems for *S*ystematic Reviews and *M*eta-*A*nalyses: The PRISMA Statement. PLoS Med 6(7): e1000097. doi:10.1371/journal.pmed1000097

For more information, visit www.prisma-statement.org.
