# Supplementary material for: Land use and pollinator dependency drives global patterns of pollen limitation in the Anthropocene
Source: Nat Commun. 2020 Aug 10;11:3999. doi: 10.1038/s41467-020-17751-y (PMC7417528; doi:10.1038/s41467-020-17751-y)
Supplement: Supplementary file 3 — Reporting Summary [file 41467_2020_17751_MOESM3_ESM.pdf]

nature research

Corresponding author(s): Joanne Bennett

Last updated by author(s): Jul 10, 2020

Reporting Summary

Nature Research wishes to improve the reproducibility of the work that we publish. This form provides structure for consistency and transparency in reporting. For further information on Nature Research policies, see our Editorial Policies and the Editorial Policy Checklist.

Statistics

For all statistical analyses, confirm that the following items are present in the figure legend, table legend, main text, or Methods section.

n/a	Confirmed	
☐	☒	The exact sample size (*n*) for each experimental group/condition, given as a discrete number and unit of measurement
☒	☐	A statement on whether measurements were taken from distinct samples or whether the same sample was measured repeatedly
☐	☒	The statistical test(s) used AND whether they are one- or two-sided<br>*Only common tests should be described solely by name; describe more complex techniques in the Methods section.*
☐	☒	A description of all covariates tested
☐	☒	A description of any assumptions or corrections, such as tests of normality and adjustment for multiple comparisons
☐	☒	A full description of the statistical parameters including central tendency (e.g. means) or other basic estimates (e.g. regression coefficient) AND variation (e.g. standard deviation) or associated estimates of uncertainty (e.g. confidence intervals)
☐	☒	For null hypothesis testing, the test statistic (e.g. F, t, r) with confidence intervals, effect sizes, degrees of freedom and P value noted<br>*Give P values as exact values whenever suitable.*
☒	☐	For Bayesian analysis, information on the choice of priors and Markov chain Monte Carlo settings
☐	☒	For hierarchical and complex designs, identification of the appropriate level for tests and full reporting of outcomes
☐	☒	Estimates of effect sizes (e.g. Cohen's *d*, Pearson's *r*), indicating how they were calculated

Our web collection on statistics for biologists contains articles on many of the points above.

Software and code

Policy information about availability of computer code

Data collection	No software was used in data collection
Data analysis	Code availability: The associated analysis code and complementary functional and ecological data are archived on github (https://github.com/idiv-biodiversity/pollen-limitation-land-use). We conducted phylogenetic mixed-effects meta-analyses using the metafor package version 2.4-0 in R version 3.6.3.

For manuscripts utilizing custom algorithms or software that are central to the research but not yet described in published literature, software must be made available to editors and reviewers. We strongly encourage code deposition in a community repository (e.g. GitHub). See the Nature Research guidelines for submitting code & software for further information.

Data

Policy information about availability of data

All manuscripts must include a data availability statement. This statement should provide the following information, where applicable:

- Accession codes, unique identifiers, or web links for publicly available datasets
- A list of figures that have associated raw data
- A description of any restrictions on data availability

Data availability: The GloPl dataset is published in scientific data doi:10.1038/sdata.2018.249 and publicly available in the Dryad repository doi:10.5061/dryad.dt437. The associated analysis code and complementary functional and ecological data are archived on github (https://github.com/idiv-biodiversity/pollen-limitation-land-use). The Land-Use Harmonization (LUH2)38 is publicly available and published on-line (https://luh.umd.edu/).

Field-specific reporting

Please select the one below that is the best fit for your research. If you are not sure, read the appropriate sections before making your selection.

☐ Life sciences ☐ Behavioural & social sciences ☒ Ecological, evolutionary & environmental sciences

For a reference copy of the document with all sections, see nature.com/documents/nr-reporting-summary-flat.pdf

Ecological, evolutionary & environmental sciences study design

All studies must disclose on these points even when the disclosure is negative.

Study description	We conducted a phylogenetic mixed-effects meta-analyses with pollen limitation as the response variable and the interaction between land use, and three plant traits that relate to their level of dependence on pollinators (pollinator dependency, and ecological and functional specialisation on pollinators.
Research sample	Experimental estimates of pollen limitation in 2247 study populations for 1247 plant species extracted from the GloPL database.
Sampling strategy	All populations under wild conditions (i.e. those not under experimental manipulation) in the GloPL database were used.
Data collection	We used data from the GloPl dataset, which is published in scientific data doi:10.1038/sdata.2018.249 and publicly available in the Dryad repository doi:10.5061/dryad.dt437. The GloPl dataset synthesis data from ~1000 published studies, where a pollen supplementation experiment was conducted. The data in GloPL was collected by the authors of this study.
Timing and spatial scale	GloPL contains all data from published pollen limitation experiments at the time the systematic literature review began, this spanned the period from 1981 up to and including 2015.
Data exclusions	In this study, we used all populations in the GloPL dataset under wild conditions to determine how land-use effects pollen limitation. Therefore we excluded cases in GloPl were an additional experimental manipulation was applied (e.g. nutrient addition, simulated herbivore) or when the plant was known to have been damaged by the supplementation experiment.
Reproducibility	The meta-analysis code and dataset is published on-line in a git repository https://github.com/idiv-biodiversity/pollen-limitation-land-use). The methods used to develop the GloPL dataset can be repeated using the methods outlined in the data descriptor published in scientific data doi:10.1038/sdata.2018.249
Randomization	NA: This is a meta-analysis
Blinding	NA: We performed a meta-analysis using published data.

Did the study involve field work? ☐ Yes ☒ No

Reporting for specific materials, systems and methods

We require information from authors about some types of materials, experimental systems and methods used in many studies. Here, indicate whether each material, system or method listed is relevant to your study. If you are not sure if a list item applies to your research, read the appropriate section before selecting a response.

Materials & experimental systems

n/a	Involved in the study
☒	☐ Antibodies
☒	☐ Eukaryotic cell lines
☒	☐ Palaeontology and archaeology
☒	☐ Animals and other organisms
☒	☐ Human research participants
☒	☐ Clinical data
☒	☐ Dual use research of concern

Methods

n/a	Involved in the study
☒	☐ ChIP-seq
☒	☐ Flow cytometry
☒	☐ MRI-based neuroimaging
